# Intrusion Detection in IoT Using Deep Learning

**DOI:** 10.3390/s22218417

**Published:** 2022-11-02

**Authors:** Alaa Mohammed Banaamah, Iftikhar Ahmad

**Affiliations:** Department of Information Technology, Faculty of Computing and Information Technology, King Abdulaziz University, Jeddah 21589, Saudi Arabia

**Keywords:** intrusion detection, internet of things, deep learning, convolutional neural network, long short-term memory, gated recurrent unit, accuracy

## Abstract

Cybersecurity has been widely used in various applications, such as intelligent industrial systems, homes, personal devices, and cars, and has led to innovative developments that continue to face challenges in solving problems related to security methods for IoT devices. Effective security methods, such as deep learning for intrusion detection, have been introduced. Recent research has focused on improving deep learning algorithms for improved security in IoT. This research explores intrusion detection methods implemented using deep learning, compares the performance of different deep learning methods, and identifies the best method for implementing intrusion detection in IoT. This research is conducted using deep learning models based on convolutional neural networks (CNNs), long short-term memory (LSTM), and gated recurrent units (GRUs). A standard dataset for intrusion detection in IoT is considered to evaluate the proposed model. Finally, the empirical results are analyzed and compared with the existing approaches for intrusion detection in IoT. The proposed method seemed to have the highest accuracy compared to the existing methods.

## 1. Introduction

Cybersecurity is one of the most challenging research topics in information technology [1,2]. It is particularly difficult to achieve when emerging technologies, such as the Internet of Things (IoT), are involved. The internet of devices is estimated to grow to 50 billion by 2020 due to its proliferation in many upcoming applications, such as smart cities, smart homes, smart cars, and intelligent industrial systems [3,4]. This growth presents a huge risk to data privacy, integrity, and availability, which may be exploited by malicious actors. Cybersecurity is not just about protecting networks and systems from unauthorized access but also safeguarding data and privacy. In recent years, there has been an increasing focus on IoT security as many new applications that rely on connected devices are being developed [5,6].

With the growing popularity of IoT, attacks against connected devices have become a critical issue. IoT devices are vulnerable to attacks in many ways, such as denial of service, eavesdropping, and privilege escalation [7]. As a result, the need to protect IoT devices from these attacks is becoming increasingly important. [8]. In addition, IoT devices are physically distributed, thus causing unauthorized access to be easy [9]. Furthermore, various devices in such an integrated system rely on wireless networks for real-time communication, which is open to eavesdropping; thus, the system is exposed to cyber threats, including web injection, that could lead to the leakage of private information and data tampering [10]. Improved and highly resilient intrusion detection systems are needed for IoT devices. Deep learning can rapidly analyze large quantities of data and support automatic adjustments of security systems upon the detection of malware or security breaches while using low computational power [11,12,13]. Security systems built on deep learning do not need a network connection for threat detection and they operate across the devices, underlying operating systems, and files [14].

The selection of a suitable deep learning method in IoT can greatly help in intrusion detection [15,16]. Such selection can be performed by comparing methods to determine the most accurate one and then implementing the selected approach. This research has many benefits, such as improving accuracy and reducing the false alarm rate of intrusion detection by using deep learning methods. It can also positively affect human lives, the economy, technology, and the environment of IoT by strengthening its security [17].

To address the above mentioned issues, a method of intrusion detection is proposed and implemented using deep learning, such as convolutional neural networks (CNNs), long short-term memory (LSTM), and gated recurrent units (GRUs). A standard dataset for intrusion detection in IoT is considered to evaluate the proposed model. The empirical findings are analyzed and compared with current IoT intrusion detection methods.

The rest of the paper is organized as follows. Section 2 describes related work. The description of the architectural model is presented in Section 3. The results are analyzed, compared, and discussed in Section 4. Section 5 concludes the paper and Section 6 presents the future direction of this work.

## 2. Related Work

IoT devices have grown rapidly and communication between these devices may pose serious risks, such as network traffic over the Internet of Things networks [18,19,20]. Network spoofing attacks, Denial of Service (DoS) attacks, and distributed denial of service (DDoS) attacks are some of the threats that can be used against the Internet of Things.

Many studies have improved IoT security and protection by using DL for increased accuracy and efficiency of the detection of security threats in IoT and to prevent them before they cause any harm. This section reviews some studies that used IDS and DL techniques in IoT. 

Susilo et al. [21] proposed an intrusion detection method using DL for IoT and found that with the increase in IoT devices the security risk and vulnerability increase as well. This study used DL techniques such as CNN. The authors performed a comparative analysis between CNN and other machine learning algorithms, such as random forest (RF) and multi-layer perceptron (MLP), by using the Bot-IoT dataset. In the experiment, CNN achieved the highest accuracy of 91.27% at a batch size of 128 and with 50 epochs; the elapsed time was 54 min and 27 s. The lowest accuracy was 88.30% at a batch size of 32 with 50 epochs; the elapsed time was 227 min and 21 s. By increasing the batch size, accuracy increased as well. The proposed model’s accuracy was lower than that of RF, which achieved 100% accuracy in DDoS and DOS attacks. The accuracy decreased when batch sizes of 32 and 64 were used. Therefore, a model of intrusion detection for IoT is required, for it could increase accuracy and reduce false alarms.

The authors in [22] explored security detection against adversarial attacks by using DL techniques, namely a self-normalizing neural network (SNN) and feedforward neural network (FNN), because the traditional method has been proven to be insufficient and useless against such attacks. They used the Bot-IoT dataset. The experiment showed that the highest accuracy achieved for FNN was 95.1% and the average precision, recall, and F1 score reached 0.95%. However, SNN 9% was found to be more resilient than FNN in terms of feature normalization to adversarial attacks. However, the Bot-IoT dataset’s feature normalization improved the resilience but affected the accuracy of SNN, which decreased to below 50%; this value is considered unsuitable for real-world protection demands. The authors in [23] proposed a novel intrusion detection and traffic analysis scheme in a network using FNN. They performed a comparative analysis between FNN and support vector classifier (SVC) by using the Bot-IoT dataset. The experiment results showed that the FNN model achieved the highest accuracy of 99.414% in multi-class classification for DDoS/DoS attacks and 0.99% across all evaluation measures: accuracy, precision, recall, and F1 score. However, the proposed solution was less precise in protecting against keylogging attacks and information theft in binary classification. In addition, the multi-class classification achieved a low accuracy of 88.9%.

Alkadi et al. [24] proposed a hybrid DL approach that used bidirectional LSTM (BiLSTM) and a blockchain based on the deep blockchain framework (DBF) to secure privacy and detect malicious activities. They analyzed the method’s accuracy and compared it with other machine learning algorithms, such as naive Bayes (NB), RF, mixture localization-based outlier (MLO), and support vector machine (SVM). They used the Bot-IoT and UNSW-NB15 datasets. The experiment showed that the highest accuracy achieved was 98.91%, and the detection rate was 99.79% in the Bot-IoT dataset. The highest accuracy reached was 99.41% and the detection rate was 99.95% in the UNSW-NB15 dataset. The limitation of the proposed solution is that IDS’ performance degrades under heavy network traffic and underperforms in alarming against and detecting a complex attack.

According to [25], IoT security usually detects attacks on either the device’s side or the cloud’s side, thereby limiting the capability to identify malicious attacks, such as botnet, phishing, and DDOS in distributed IoT devices. The authors in [18] introduced cloud-based detection using DL approaches, such as distributed CNN (DCNN) for IoT devices and LSTM for back-end hosts in the cloud. The two models detect attacks on both sides. Their accuracy was analyzed using the N_BaIoT dataset. The experiment showed that the highest accuracy achieved in LSTM was 0.9784% at the back end, the precision reached 0.9781%, the recall was 0.9500%, the F-score was 0.9625%, FPR was 0.0001%, and TPR was 0.9999%. However, the proposed solution could not detect emerging attacks, which still leaves IoT devices vulnerable to risks.

Samy et al. [26] discussed the importance of the risk of having an increased number of devices connected to IoT, especially zero-day attacks. The authors proposed a framework that uses an LSTM DL model to detect unknown attacks. They compared it with other DL models, such as GRU, LSTM, CNN, CNN-LSTM, and DNN, in five different IoT datasets. The experiment showed that the highest accuracy achieved by LSTM was 99.96% in binary classification and 99.65% in multi-class classification, with a 99.97% detection rate. However, the proposed model needs massive datasets and a long time to train. The authors in [27] discussed the risk posed by network traffic over IoT networks. This study used machine learning methods, such as Hoeffding tree (HT) and naive Bayes, and a DL method (DNN). They used four different IoT datasets. The experiment showed that the highest accuracy achieved by DNN was 0.9975% in binary classification with seven hidden layers. The highest precision, recall, and F score were 0.9937%, 0.9937%, and 0.9937%, respectively. However, the experiment tested only four different attacks (scanning, DoS, MITM, and Mirai), which are not enough to represent real-world attacks. According to [28], DL methods exhibit extensive performance but have a prominent drawback: they need massive data for training algorithms. This study used two methods: LSTM and ensemble learning. A comparative analysis was performed between LSTM and other machine learning approaches, such as RF, stacking, bagging, AdaBoost, and XGBoost, by using Smart-Fall datasets. The experiment showed that the highest accuracy achieved in LSTM was 0.934%; the precision reached 0.920%, the recall was 0.934%, and the F score was 0.9178%. The highest accuracy achieved in RF was 0.999%. The accuracy of LSTM was lower than that of other methods and techniques. However, the study applied the method on only one dataset for an evaluation, which is considered a limitation. 

Shobana and Poonkuzhali [29] introduced a novel approach to detect IoT malicious attacks by using system calls and RNN. They employed the IOTPOT dataset. The experiment showed that the highest accuracy achieved was 98.712% with four epochs and a single hidden layer, and the error rate was 1.288%. However, this study could still be improved by implementing multiclass classification and category malware on system calls. It could also be enhanced by applying other DL methods, such as LSTM. The literature review is summarized in Table 1.

One of the limitations of using deep learning in security enhancement within IoT traffic is balancing between high accuracy and minimal false alarms during communication. This limitation mainly presents in the CNN. Additionally, using feed-forward neural networks (FNN) for multi-class classification is a limiting factor for the protection of the IoT network against information theft and key logging, which is only effective in binary classification approaches. The third limitation of the proposed framework is the degradation in performance of intrusion detection systems (IDS) whenever the network is under heavy traffic load. In cases of detecting complex attacks, the framework underperforms, which mainly happens when using a Bidirectional Long Short-Term Memory (BiLSTM) classifier. Lastly, using a Deep Neural Network (DNN) results in an increase in the execution time as the training dataset size increases. All these limitations result in lower accuracy and increased false alarm rates, which becomes a general problem in IoT networks. Therefore, an intrusion detection model is essential that can overcome the abovementioned issues. Thus, deep learning models including convolutional neural networks (CNNs), long short-term memory (LSTM), and gated recurrent units are proposed to improve the accuracy.

## 3. Material and Methods

The implementation of this research will follow a stepwise methodology that involves using a deep learning model to develop a comprehensive IoT security model that improves the accuracy of detecting security threats.

**Figure 1:**
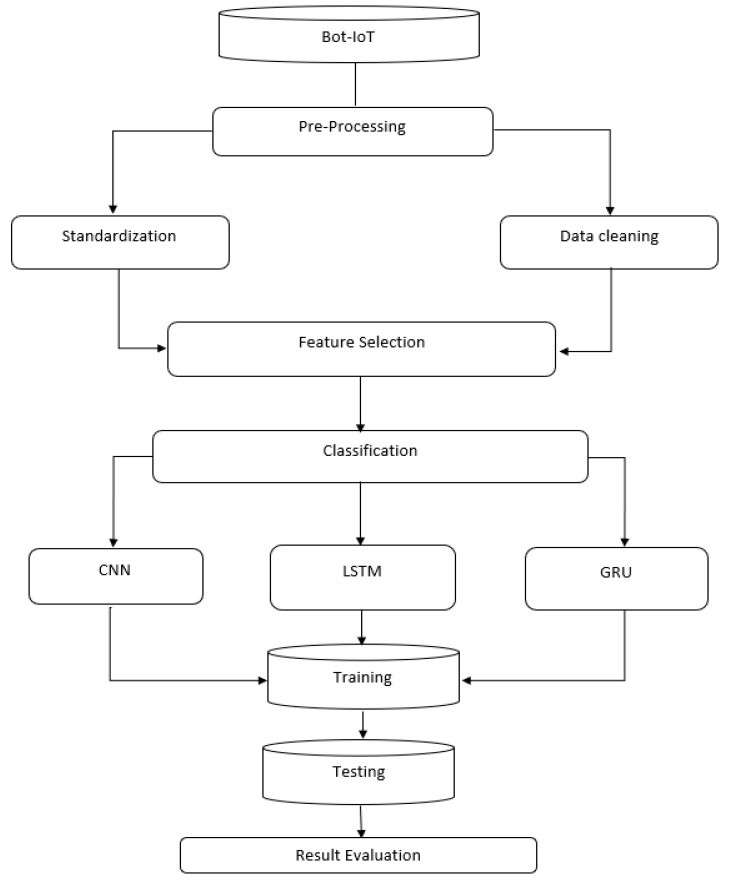
Architecture model.

The model is shown in Figure 1. The first part of the figure shows the preprocessing of the datasets. The preprocessing consisted of three sub-steps: scaling, normalization, and data cleaning. Then, the dataset was labeled. The next classification step was performed using CNN, LSTM, and GRU. Afterward, we trained, tested, and evaluated our model.

### 3.1. Step 1: Bot-IoT

This dataset was developed on a realistic network design with traffic from botnets and normal systems [30,31]. Attacks are categorized and thereafter labeled. Botnet traffic is created by compromised network nodes/bots that receive commands from a central node called the botmaster. Botnet traffic can be used to attack a system sending back information to the botmaster. In an IoT environment, traffic is sent over a publish and subscribe communication protocol implemented over TCP/IP [32,33,34].

### 3.2. Step 2: Pre-Processing of the Datasets

In this step, the raw dataset is processed and made suitable for a DL method. This procedure includes standardization, normalization, and data cleaning [35]. The step is divided into three sub-steps. The first sub-step is dataset standardization. This step is important because it ensures that the data are in the same scale and have a distribution value between 0 and 1 based on the standard normal distribution. The second sub-step is data normalization. Normalization consists of transforming the data. This step is important to avoid negative values, which are unacceptable to neural networks. We normalized all the data in the dataset between 0 and 1. The third step, data cleaning, consists of removing unwanted data, such as NaN and null values.

### 3.3. Step 3: Feature Selection

In this step, the best features are selected for the model. This step is important in DL because it affects the performance of the model. If we use an inappropriate set of features, the results of the model will be poor. Thus, in this step, we selected the features to be used by our model. We adopted four features for Bot-IoT. The features were “dur”, “rate”, “srate”, and “drate”, and these features were used to represent time and duration that affect the classification of attacks.

### 3.4. Step 4: Classification

This step adopts different models to predict the attack. We used three different types of neural networks to classify the attacks. These NNs were CNN, LSTM, and GRU. We employed TensorFlow and Kera to implement CNN, GRU, and LSTM and Python to implement the neural network models.

### 3.5. Step 5: Trained, Tested, and Evaluated

We trained the models with the selected features. In our model, we used 80% of the data for training and the remaining 20% for testing. Therefore, we could train and test the model with only 20% of the dataset. This allowed us to accurately predict the attack.

## 4. Results

This section contains experimental results implemented in Collaboratory by Google Research. All experiments were performed using the Python programming language. The datasets were divided into training and testing datasets for the experiments. The Bot-IoT dataset was divided into training and test sets 80%–20%, respectively. The number of samples in the test set for each dataset was equal to the size of the dataset. We used the Kera’s library to build our classifiers and we used TensorFlow as the backend of the Kera’s library. Table 2 describes training parameters of CNN. LSTM and GRU classifier training parameters are mentioned in Table 3.

The results of the experiments in Table 4 below shows the results of the different classifiers in terms of accuracy and false alarm when applied to the Bot-IoT dataset. As we can see from the table, the LSTM accuracy rate is the highest compared to CNN and GRU. Table 5 presents precision, recall, and F1 score of CNN, LSTM, and GRU.

**Figure 2:**
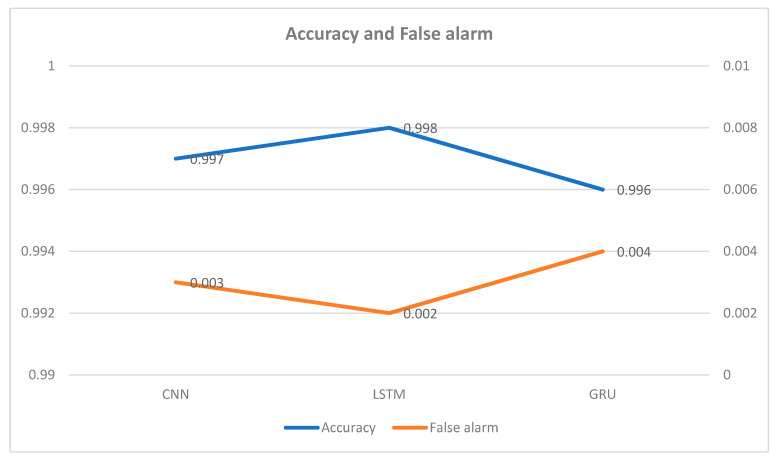
Experiment results for accuracy and false alarm.

**Figure 3:**
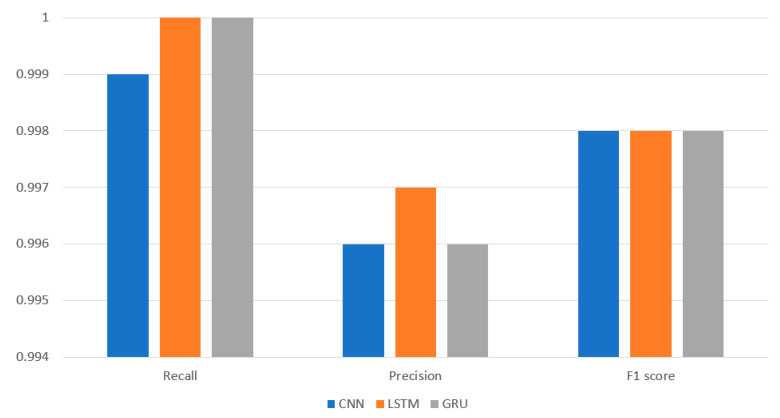
Experiment results for precision, recall and F1 score.

Figure 2 shows accuracy and false alarm among the CNN, LSTM, and GRU. The LSTM outperforms the CNN and GRU in accuracy which is 99.8% as compared to CNN and GRU. Figure 3 depicts the F1 score, recall, and precision for CNN, LSTM, and GRU. In regard to precision, the LSTM performs better than the CNN and GRU.

We compared the performance of our proposed model with other state-of-the-art methods. The results are shown in Table 6.

**Figure 4:**
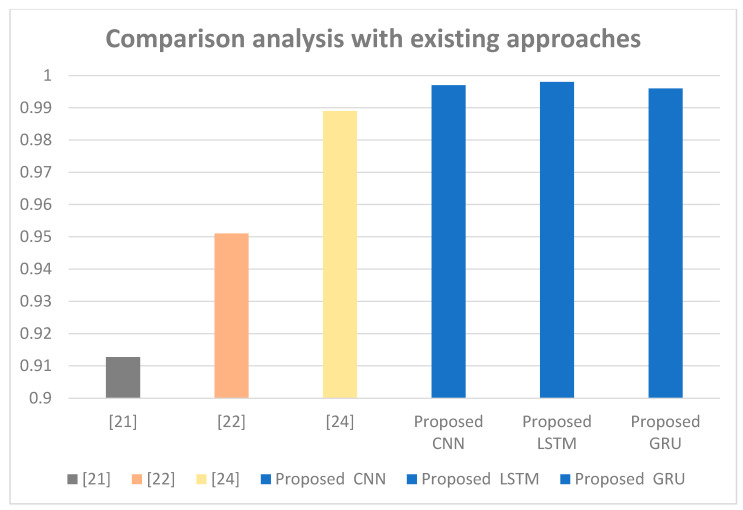
Accuracy comparison with existing approaches.

The experimental results for the three classifiers CNN, LSTM, and GRU are compared with existing state-of-the-art approaches. Figure 4 shows that our approach is effective compared to the existing state-of-the-art approach. In the Bot-IoT dataset, the proposed approach achieved the highest accuracy of 99.8%.

## 5. Conclusions

In this paper, we presented a study on the use of deep learning methods in detecting intrusions in IoT devices. In our work, we have used a standard dataset Bot-IoT for intrusion detection in IoT. We have also used different types of Deep Learning methods such as the Convolutional Neural Network, Gated Recurrent Unit, and Long Short Memory Neural Network for intrusion detection in IoT. We have evaluated the proposed model and compared it with existing approaches. The experimental results have shown that the proposed method can be effective for intrusion detection in IoT.

## 6. Future Works

We will explore more datasets for intrusion detection on IoT devices in future. Recently, some new IoT datasets have been made available. This work can also be extended to study other variants of classifiers such as genetic algorithm (GA) and bidirectional short-term memory (BiLSTM) for better performance. 

## Figures and Tables

**Table 1:** Summary of Literature Review.

Ref.	Classifier	Dataset	Accuracy and Performance	Limitation
[21]	Convolutional neural network (CNN)	Bot-IoT	The accuracy achieved 91.27% in batch size 128 and the lowest 88.30% in batch size 32.	The accuracy decreases when using 32 and 64 batch size.
[22]	Feedforward Neural Networks (FNN)	Bot-IoT	The accuracy achieved 95.1%, and the average precision, recall, and F1-score reached 95%.	The Bot-IoT dataset’s feature normalization shows that the accuracy would drop below 50%.
[23]	Feed-forward neural networks (FNN)	Bot-IoT	The accuracy achieved 99.414% in multi-class classification for DDoS/DoS attacks and 99% across all evaluation measures: accuracy, precision, recall, and F1 score.	The proposed solution has proved to be less precise in protecting against keylogging attacks and information theft in binary classification, also, the multi-class classification has achieved the low accuracy of 88.9%.
[24]	Bidirectional Long Short-Term Memory (BiLSTM)	Bot-IoT and UNSW-NB15	The accuracy achieved 98.91 %, the detection rate achieved 99.79% in the Bot-IoT dataset, the accuracy has reached 99.41%, and the detection rate achieved 99.95% in the UNSW-NB15 dataset.	The proposed solution’s limitation is that IDS’ performance degrades under heavy network traffic and underperforms in alarming against and detecting a complex attack.
[25]	Long short-term memory model (LSTM)	N_BaIoT	The accuracy achieved 97.84% at the back-end level and precision achieved 97.81%, recall 95%, F-score 96.25%, FPR 0.0001, and TPR 0.9999	The proposed solution does not function on detecting emerging attacks.
[26]	Long short-term memory model (LSTM)	N_BaIoT-2018 , CICIDS-2017, RPLNIDS-2017 and NSL-KDD	The accuracy achieved in binary classification 99.85% in N_BaIoT 2018 dataset and precision achieved 98.64%, recalled 99.81%, F-score 99.12%, FPR 0.1%, and DR 99.81%.	The proposed model needs massive datasets and longer time to train.
[27]	Deep Neural Network (DNN)	4 datasets of IoT	The accuracy achieved in binary classification 99.75% with seven hidden layers and precision achieved 99.37%, recalled 99.37%, and F-score 99.37%	The experiment tests on only four different attacks (Scanning, DoS, MITM, and Mirai).
[28]	Long short-term memory model (LSTM) Additionally, Random Forest (RF)	SmartFall dataset	The accuracy achieved in LSTM is 93.4% and precision achieved 92%, recalled 93.4%, and F-score 91.78%. The accuracy achieved in RF 99.9%. Additionally, precision achieved 99.9%, recalled 99.9%, and F-score 99.9%.	The accuracy of LSTM is considered low compared with other methods and techniques. The study applied only one dataset as an evaluation.
[29]	Recurrent neural network (RNN)	IOTPOT	The accuracy achieved is 98.712%. Additionally, error rate is 1.288%.	It could be improved by implementing a multiclass classification and category malware on system calls. It could also be enhanced by applying other deep learning methods, such as LSTM.

**Table 2:** CNN classifier training parameters for Bot-IoT.

Number	Parameter	Explanation
1	Classifier	CNN
2	Layers	(4) Input (10) Hidden and (1) Output
3	Input Features	4
4	Output	Normal (0) Attack (1)
5	Training Dataset	80% for Training 20% for Testing

**Table 3:** LSTM and GRU classifier training parameters for Bot-IoT.

Number	Parameter	Explanation
1	Classifier	LSTM Additionally, GRU
2	Layers	(4) Input (100) Hidden and (1) Output
3	Input Features	4
4	Output	Normal (0) Attack (1)
5	Training Dataset	80% for Training 20% for Testing

**Table 4:** Result experiment for accuracy and false alarm.

Classifier	Dataset	Accuracy	FA
CNN	Bot-IoT	0.997	0.003
LSTM	Bot-IoT	0.998	0.002
GRU	Bot-IoT	0.996	0.004

**Table 5:** Result experiment for precision, recall, and F1 score.

Classifier	Dataset	Precision	Recall	F1 Score
CNN	Bot-IoT	0.996	0.999	0.998
LSTM	Bot-IoT	0.997	1.000	0.998
GRU	Bot-IoT	0.996	1.000	0.998

**Table 6:** Model comparison with other state-of-the-art methods.

Ref	Classifier	Dataset	Accuracy and Performance
[21]	Convolutional Neural Network (CNN)	Bot-IoT	The accuracy achieves 91.27% in batch size 128 and the lowest 88.30% in batch size 32.
[22]	Feedforward Neural Networks (FNN)	Bot-IoT	The accuracy achieves 95.1%, and the average precision, recall, and F1-score reached 0.95%
[24]	Bidirectional Long Short-Term Memory (BiLSTM)	Bot-IoT	The accuracy achieves 98.91 %, and the detection rate achieved 99.79%
Proposed model	Convolutional Neural Network (CNN)	Bot-IoT	The accuracy achieves 99.7% and precision achieve 99.6%, recall 99.9%, and F-score and detection rate 99.8%
Proposed model	Long Short-Term Memory (LSTM)	Bot-IoT	The accuracy achieves 99.8%, precision achieve 99.7%, recall 100%, and F-score and detection rate 99.8%
Proposed model	Gated Recurrent Unit (GRU)	Bot-IoT	The accuracy achieves 99.6% and precision achieve 99.6%, recall 100%, and F-score and detection rate 99.8%

## Data Availability

Not applicable.

## References

[1] Zhang J., Pan L., Han Q.-L., Chen C., Wen S., Xiang Y. (2021). Deep learning-based attack detection for cyber-physical system cybersecurity: A survey. IEEE/CAA J. Autom. Sin..

[2] Lee I. (2020). Internet of Things (IoT) cybersecurity: Literature review and IoT cyber risk management. Futur. Internet.

[3] Almiani M., AbuGhazleh A., Al-Rahayfeh A., Atiewi S., Razaque A. (2020). Deep recurrent neural network for IoT intrusion detection system. Simul. Model. Pract. Theory.

[4] Azumah S.W., Elsayed N., Adewopo V., Zaghloul Z.S., Li C. A deep lstm based approach for intrusion detection iot devices network in smart home. Proceedings of the 2021 IEEE 7th World Forum on Internet of Things (WF-IoT).

[5] Thakkar A., Lohiya R. (2021). A review on machine learning and deep learning perspectives of IDS for IoT: Recent updates, security issues, and challenges. Arch. Comput. Methods Eng..

[6] Li Y., Zuo Y., Song H., Lv Z. (2021). Deep learning in security of internet of things. IEEE Internet Things J..

[7] Idrissi I., Boukabous M., Azizi M., Moussaoui O., El Fadili H. (2021). Toward a deep learning-based intrusion detection system for IoT against botnet attacks. IAES Int. J. Artif. Intell. (IJ-AI).

[8] Venkatraman S., Surendiran B. (2019). Adaptive hybrid intrusion detection system for crowd sourced multimedia internet of things systems. Multimedia Tools Appl..

[9] Alladi T., Chamola V., Sikdar B., Choo K.-K.R. (2020). Consumer IoT: Security vulnerability case studies and solutions. IEEE Consum. Electron. Mag..

[10] Asharf J., Moustafa N., Khurshid H., Debie E., Haider W., Wahab A. (2020). A Review of Intrusion Detection Systems Using Machine and Deep Learning in Internet of Things: Challenges, Solutions and Future Directions. Electronics.

[11] Wang X., Zhao Y., Pourpanah F. (2020). Recent advances in deep learning. Int. J. Mach. Learn. Cybern..

[12] Abu Al-Haija Q., Zein-Sabatto S. (2020). An efficient deep-learning-based detection and classification system for cyber-attacks in IoT communication networks. Electronics.

[13] Abu Al-Haija Q., Al-Dala’ien M.A. (2022). ELBA-IoT: An Ensemble Learning Model for Botnet Attack Detection in IoT Networks. J. Sens. Actuator Netw..

[14] (2020). Pioneering Deep Learning in the Cyber Security Space: The New Standard?. Information Age.

[15] Khan T., Sarkar R., Mollah A.F. (2021). Deep learning approaches to scene text detection: A comprehensive review. Artif. Intell. Rev..

[16] Aversano L., Bernardi M.L., Cimitile M., Pecori R. (2021). A systematic review on Deep Learning approaches for IoT security. Comput. Sci. Rev..

[17] Stefanos T., Lagkas T., Rantos K. (2022). Deep learning in iot intrusion detection. J. Netw. Syst. Manag..

[18] Davis B.D., Mason J.C., Anwar M. (2020). Mason, and Mohd Anwar. Vulnerability studies and security postures of IoT devices: A smart home case study. IEEE Internet Things J..

[19] Jiang X., Lora M., Chattopadhyay S. (2020). An experimental analysis of security vulnerabilities in industrial IoT devices. ACM Trans. Internet Technol..

[20] Chanal P.M., Kakkasageri M.S. (2020). Kakkasageri. Security and privacy in IOT: A survey. Wirel. Pers. Commun..

[21] Susilo B., Sari R.F. (2020). Intrusion Detection in IoT Networks Using Deep Learning Algorithm. Information.

[22] Ibitoye O., Shafiq O., Matrawy A. Analyzing Adversarial Attacks against Deep Learning for Intrusion Detection in IoT Networks. Proceedings of the 2019 IEEE Global Communications Conference (GLOBECOM).

[23] Ge M., Fu X., Syed N., Baig Z., Teo G., Robles-Kelly A. Deep Learning-Based Intrusion Detection for IoT Networks. Proceedings of the 2019 IEEE 24th Pacific Rim International Symposium on Dependable Computing (PRDC).

[24] Alkadi O., Moustafa N., Turnbull B., Choo K.-K.R. (2020). A Deep Blockchain Framework-enabled Collaborative Intrusion Detection for Protecting IoT and Cloud Networks. IEEE Internet Things J..

[25] Parra G.D.L.T., Rad P., Choo K.-K.R., Beebe N. (2020). Detecting Internet of Things attacks using distributed deep learning. J. Netw. Comput. Appl..

[26] Samy A., Yu H., Zhang H. (2020). Fog-Based Attack Detection Framework for Internet of Things Using Deep Learning. IEEE Access.

[27] Pecori R., Tayebi A., Vannucci A., Veltri L. IoT Attack Detection with Deep Learning Analysis. Proceedings of the 2020 International Joint Conference on Neural Networks (IJCNN).

[28] Farsi M. (2021). Application of ensemble RNN deep neural network to the fall detection through IoT environment. Alex. Eng. J..

[29] Shobana M., Poonkuzhali S. A novel approach to detect IoT malware by system calls using Deep learning techniques. Proceedings of the 2020 International Conference on Innovative Trends in Information Technology (ICITIIT).

[30] Koroniotis N., Moustafa N., Sitnikova E., Turnbull B. (2019). Towards the development of realistic botnet dataset in the internet of things for network forensic analytics: Bot-iot dataset. Future Gener. Comput. Syst..

[31] Koroniotis N., Moustafa N., Sitnikova E., Slay J. (2017). Towards developing network forensic mechanism for botnet activities in the IoT based on machine learning techniques. Proceedings of the International Conference on Mobile Networks and Management.

[32] Koroniotis N. (2020). Designing an effective network forensic framework for the investigation of botnets in the Internet of Things. Ph.D. Dissertation.

[33] Koroniotis N., Moustafa N., Schiliro F., Gauravaram P., Janicke H. (2020). A holistic review of cybersecurity and reliability perspectives in smart airports. IEEE Access.

[34] Koroniotis N., Moustafa N. (2020). Enhancing network forensics with particle swarm and deep learning: The particle deep framework. arXiv.

[35] Peterson J.M., Leevy J.L., Khoshgoftaar T.M. A review and analysis of the bot-iot dataset. Proceedings of the 2021 IEEE International Conference on Service-Oriented System Engineering (SOSE).

